# Supplementary material for: Content-rich biological network constructed by mining PubMed abstracts
Source: BMC Bioinformatics. 2004 Oct 8;5:147. doi: 10.1186/1471-2105-5-147 (PMC528731; doi:10.1186/1471-2105-5-147)
Supplement: Additional File 5 — The original Chilibot query results of the term "long-term potentiation (LTP)" and 22 other terms, limiting the latest references analyzed to the years 1990, 1995, 2000, and 2004. [file 1471-2105-5-147-S5.bz2 › chilibotAdditionalFile5/ltp2004/html/PKA_ATF.html]

 


 **PKA** and **ATF** 
  
Found 35 abstracts in PubMed,  **30 abstracts were retrieved and analyzed**.  


---

 Search Google  |
 PDF files only 
|  EDU domain only 

---

**Interactive relationship** (e.g. stimulation, inhibition, etc)

- Our results confirm that the activation of the HTLV 1 promoter through Tax and factors of the  **ATF**  CREB family is  **PKA**  independent.  Ref: 9733879 J Virol, 1998
- ERK activation by the  **PKA**  pathway was not required for CREB  **ATF**  1 phosphorylation but rather was necessary for CREB dependent up regulation of C EBPs expression.  Ref: 11682632 Mol Endocrinol, 2001
- In fission yeast, an  **ATF**  CREB family transcription factor Atf1 Pcr1 plays important roles in the activation of early meiotic processes via the stress activated protein kinase SAPK and the cAMP dependent protein kinase  **PKA**  pathways.  Ref: 11779789 Genetics, 2001
- Collectively, these results indicate the DSE are TRE like cAMP responsive elements that bind both AP 1 and CREB  **ATF**  permitting induction of the HIV 1 LTR by both PKC and  **PKA**  activation signals.  Ref: 9201233 Virology, 1997
- CREB 2 is an unconventional member of the  **ATF**  CREB family in that it lacks a protein kinase A  **PKA**  phosphorylation site and has been reported to negatively regulate transcription from the cyclic AMP response element of the human enkephalin promoter.  Ref: 9733879 J Virol, 1998
- Accumulation of steady state level of  **ATF**  2 was observed and was also modulated by treatment with H 89, a  **PKA**  inhibitor.  Ref: 9310369 Eur J Biochem, 1997

**Parallel relationship** (e.g. studied together, co-existance, homology, etc.)

- DSE binding complexes in nuclear protein extracta from colonic epithelial cells are shown to contain  **ATF**  1,  **ATF**  2, and CREB and transfection of either an  **ATF**  2 or  **PKA**  expressing plasmid transactivated the DSE.  Ref: 9201233 Virology, 1997
- We conclude that differentiation dependent transcription of the CGalpha gene in villous trophoblasts is mainly governed by increasing expression of AP 2alpha and  **PKA**  dependent phosphorylation of CREB 1 and  **ATF**  1.  Ref: 11014230 Endocrinology, 2000
- 3 In contrast the activation of cAMP  **PKA**  mediated a significant inhibiton of the phosphorylation of ERK1 2 JNK and p38 MAPK and  **ATF**  2 but it enhanced the phosphorylation of CREB.  Ref: 12110939 Sheng Wu Hua Xue Yu Sheng Wu Wu Li Xue Bao < MedlineTA>Sheng Wu Hua Xue Yu Sheng Wu Wu Li Xue Bao , 2000
- Since enhanced HIV 1 expression is associated with activation of the cAMP dependent protein kinase A  **PKA**  signaling pathway, we determined whether binding of CREB  **ATF**  proteins to the DSE mediate cAMP  **PKA**  activation of the HIV 1 LTR.  Ref: 9201233 Virology, 1997
- In addition,  **PKA**  mediated transcriptional regulation of grp78 in OA >HS treatment is through regulation of protein phosphorylation as well as de novo synthesis of  **ATF**  2.  Ref: 9310369 Eur J Biochem, 1997
